# Estimated HIV cases and costs in South Africa due to global warming from 2000 to 2050: a modeling study

**DOI:** 10.1038/s41598-026-57530-1

**Published:** 2026-07-14

**Authors:** Sachin Silva, Gesine Meyer-Rath, J. A. Michael Reid

**Affiliations:** 1 Institute for Global Health Sciences, University of California San Francisco, 550 16th Street, San Francisco, CA 94158 USA; 2 Health Economics and Epidemiology Research Office (HE2RO), University of the Witwatersrand, Princess of Wales Terrace, Parktown, Johannesburg, 2193 South Africa; 3 Boston University School of Public Health, 715 Albany St, Boston, MA 02118 USA; 4 School of Medicine, University of California San Francisco, 513 Parnassus Avenue, San Francisco, CA 94143-0410 USA

**Keywords:** Global warming, South Africa, People living with HIV, ART, Climate sciences, Diseases, Environmental social sciences, Health care, Scientific community

## Abstract

The impact of global warming on HIV prevalence and costs in South Africa are not known. We estimated for 2000–2020 using recorded surface temperatures and for 2021–2050 using predicted temperatures from the MAGICC7.0 climate model. In the latter case we assumed that societal development and temperatures would follow the narrative of Shared Socioeconomic Pathway (SSP) 2. We considered as a best-case, pathway 1 (SSP1). To estimate the number of people whose infection status can be associated with rising temperatures (PLHIV-T), we estimated temperature-related prevalence risks, which we applied to predicted prevalence from the Thembisa HIV transmission model. We derived incidence from prevalence using previously established methods. For the former, we also estimated follow-up antiretroviral therapy (ART) costs. PLHIV-T has increased by 7-fold from 2001 to 2020, from 1,712 to 13,254. ART costs for PLHIV-T have correspondingly increased from US$2.07 million in 2010 to US$2.89 million in 2015 and then declined to US$2.02 million in 2020. If societal development remained unchanged from 2020 to 2050, PLHIV-T associated with temperatures predicted by SSP2 increases by nearly 1.5-fold by 2050, to 14,033 (0.2% of all prevalent cases; incidence at a rate of 1.44 per 100-person-years) with US$2.25 million in ART costs. Alternatively, if societal development followed SSP1, then in 2050, the penalty would be nearly the same with 14,009 instead of 14,033 PLHIV-T and US$ 2.25 million in ART costs. Societal development alone does not sufficiently mitigate climate related health losses. Stakeholders must therefore prioritize health adaptation within climate financing and integrate HIV response planning into national climate resilience strategies.

**Supplementary Information:**

The online version contains supplementary material available at 10.1038/s41598-026-57530-1.

## Introduction

In November 2024, the UN Climate Change Conference (COP29) reached a New Collective Quantified Goal on Climate Finance (NCQG) pledging to increase financing to developing countries from US$100 billion to US$300 billion by 2035. The NCQG fell short of the US$1·3 trillion needed globally^1^. COP30 held in November 2025 did little to mitigate the shortfall. The final agreement which included a pledge to triple adaptation funding, though a positive signal, delays funding to 2035 from 2030^2^.Annual climate-related losses are set to exceed the US$143 billion recorded from 2000 to 2019^3^. African countries, who contribute less than 3.8% to global greenhouse gas emissions^4^ are most vulnerable to climate events due to lower adaptive capacity stemming from modest economic diversification and high dependence on agro-ecosystems^5^. In South Africa, confronting the compounded burden of HIV, tuberculosis^6^, and malaria^7^, all of which are known to be associated with climate events^8,9^, the health consequences of inadequate investment in climate mitigation and adaptation are especially concerning.

Climate events such as rainfall^10^, flooding^11^, droughts^12^, and warming are thought to be associated with HIV transmission and noncontinuity of care^13^ via three principal pathways. Climate related disruptions of agricultural output are thought to increase transactional sex as a means to mitigate food insecurity^11^ and economic vulnerability^14^. Climate related migration is thought to give rise to new sexual networks^15^ and also cause antiretroviral therapy (ART) nonadherence, lost to follow-up, development of drug resistance and general noncontinuity of HIV care^13^. Climate events are also thought to erode public health infrastructure, especially HIV prevention and treatment services^15^. Income shocks exerted via these pathways are also thought to drive higher prevalence^14^. Among climate events, heat and droughts have exacted the heaviest toll in Africa with 64% of land area affected by at least one month of extreme drought per year. Africa also saw the highest losses in income due to heat stress in 2022, wiping out the equivalent of 4.1% of the GDP, mainly from losses in the agricultural sector^16^.

As of 2023, South Africa requires on average US$17.9 billion per year to meet its net-zero goals by 2050, and US$28.6 billion per year to meet its Nationally Determined Contributions^17^ by 2030. This necessitates a three to five-fold increase from the current annual average climate budget of US$7 billion^18^. The public sector HIV program in South Africa has been funded primarily from domestic sources since its beginning in 2004. In 2023, the President’s Emergency Plan for AIDS Relief (PEPFAR) provided 21% of South Africa’s total HIV expenditure of $1.86 billion, covering 50% of prevention programs and treatment retention activities^19^. In February 2025, following the mandated review all US foreign assistance programs, any potential funding disruptions were estimated to have far-reaching consequences with resulting excess HIV cases expected to be as high as 150 000–296 000^20–22^. These new fiscal uncertainties further underscore the importance of strengthening domestic health systems and increasing resilience to climate-related health threats.

We considered the case of HIV in South Africa where 7·8 million people - almost one in five adults, are living with HIV and the HIV treatment programme is primarily domestically funded^20,23^. We asked: What has been the climate penalty so far in terms of excess HIV cases and their treatment costs? What will be the penalty in the future if resource limitations hindered climate adaptation and mitigation action and the current climate trajectory remained unchanged? We focus on deaths directly attributable to surface temperature changes due to availability of reliable data, acknowledging that other climate-related health impacts, such as vector-borne diseases and waterborne illnesses, may also be influenced by climate change.

## Methods

To answer the former question – the climate penalty so far (from 2000 to 2020), we used recorded land temperature increases from pre-industrial levels (1850–1900), available for South Africa from the National Center for Environmental Information^24^. To answer the latter question – the climate penalty in the future (from 2021 to 2050), we assumed that in the absence of new mitigation or adaptation action, societal development would follow the narrative of Shared Socioeconomic Pathway 2 (SSP245 or SSP2) which assumes that social, economic, and technological trends would not shift markedly from historical patterns with moderate challenges to mitigation and adaptation. We also considered Shared Socioeconomic Pathway 1 (SSP119 or SSP1) to represent a best-case scenario where countries would gradually shifts towards a sustainable path, with more inclusive development that respects perceived environmental boundaries resulting in low challenges to mitigation and adaptation.As comparators, we also considered SSP370 (SSP3) which assumes that nationalism, competitiveness, and regional countries would push countries towards rivalry resulting in high challenges to mitigation and adaptation, and SSP585 (SSP5) where faith in markets, innovation, technological progress would drive a greater reliance on fossil fuels resulting in high mitigation challenges but low adaptation challenges^25,26^. For each scenario, we used temperature increases estimated by the MAGICC7.0 reduced complexity earth system model^25,27^. To estimate HIV cases and ART coverage from 2000 to 2050, we used outputs from Thembisa, a deterministic, age structured compartmental transmission model, which has been described elsewhere^28^. To estimate the share of HIV cases that can be associated with temperature increases, we extended the regression framework proposed by Baker^29^ and recalculated South Africa specific relative risks taking into account population prevalence and ART coverage predicted by the transmission model. We calculated incident HIV cases from prevalent cases using methods described by Hallett et al.^30^. When estimating the cost of ART, for the 2000 to 2025 period, we compiled per patient per year costs from literature and prior National AIDS Spending Assessment reports^31^. For the 2025 to 2050 period, we assumed the unit costs reported by Jamieson and colleagues^32^ but adjusted for inflation. We report excess HIV cases and costs from 2000 to 2020 in relation to recorded prevalence, and from 2021 to 2050, in relation to modeled prevalence. We report all monetary values in 2024 US$ rates. Sensitivity to parameter uncertainty and choice were evaluated using a Latin hypercube sampling algorithm to sample from suitable distributions^33^(appendix).

## Results

By 2000, South Africa’s land temperature had increased above pre-industrial levels by 1.11 °C. By 2020, it had increased by 1.65 °C^25^. The number of PLHIV-T associated with this temperature increase ranges between 0.05% and 0.18% (mean = 0.14%; std. deviation = 0.04%). This means that in 2001, of the 3.56 million people living with HIV (PLHIV), it is plausible that 1,712 (0.05%) (range: 1,008 − 2,415) could be associated with land temperature increase. We estimated that 0.03% of new cases (range: 0.03–0.03) can be associated with rising temperatures. In 2010, of the 5.62 million PLHIV, 9,195 (0.16%) can be associated with warming, of which 0.12% are new cases (0.06–0.18); 1,833 PLHIV-T will require follow-up first line ART at a cost of US$2.07 million (0.16% of US$1.26 billion in total follow-up ART costs). In 2020, there were 7.45 million PLHIV of which 13,254 (0.18%) (7,808 − 18,699) can be associated with global warming, including 0.15% of new cases [0.08–0.22]). Of the 13,254 individuals, 9,549 were on follow-up ART at a cost of US$2.01 million (US$1.19-US$2.84 million) (0.18% of the US$1.13 billion).

**Fig. 1:**
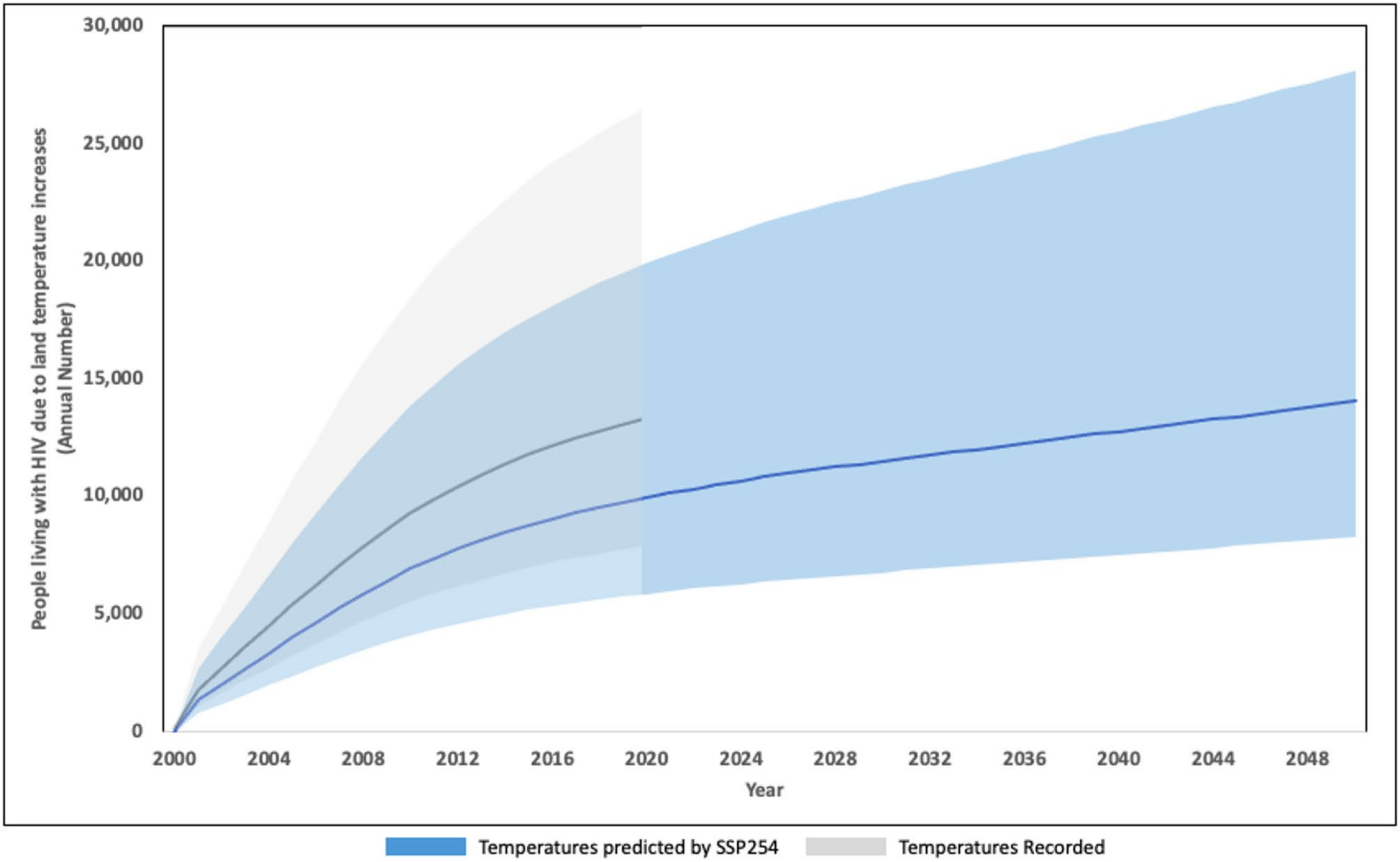
PLHIV due to land temperature increases from pre-industrial levels: by (**a**) recorded land temperatures for 2000–2020; (**b**) predicted land temperatures from SSP245 shared socioeconomic pathway for 2020–2050. Recorded temperatures are from the National Center for Environmental Information^20^. Predicted temperatures are from the MAGICC7.0 model^14^. The uncertainty bounds represent uncertainty due to the temperature dependent probability that an individual tests positive for HIV, calculated based on Baker^7^. The total number of PLHIV, which was used for the calculation, is from the Thembisa transmission model^24^.

Had temperatures between 2000 and 2020 followed the trajectory predicted by SSP245, then in 2010, of the 5.61 million PLHIV, 2,272 cases could have been averted (6,924 [4,078 − 9,768] rather than 9,195 cases due to SSP245) thus requiring an ART cost of US$1.56 million - US$511,816 less than costs incurred. Of the 6,924 PLHIV-T, 0.15% are due to new cases (0.07–0.16). By 2020, the share averted rises to 3,303 (9,951 rather than the 13,254 cases due to SSP245) thus requiring US$1.51 million in ART cost - US$502,328 less − 0.18% are new cases (0.13–0.23) (Fig. 1; Table 1).

**Table 1:** Incident and prevalent HIV cases and ART costs in South Africa due to land temperature increases from 2000 to 2050. *ART costs are not reported for 2000 as ART use was minimal. The annual number of PLHIV were derived based on previously established excess risks. The precents of PLHIV for each year and scenario represent percent of annual accrued total PLHIV. For 2000, the temperature increases from 1999 was used as the baseline. For subsequent years, temperature in 2000 was used as the baseline. Annual incident infections were derived based on prevalent infections using methods described by Hallett et al. for mature/stable epidemics which we assumed South Africa to be^25^. The range corresponds to the uncertainty in the relative risks derived based on the framework reported by Baker^29^. Total annual incident infections are reported in appendix Table 4. ART unit costs were sourced from published sources.The averted (%) represents the difference in PLHIV-T calculated based on observed temperatures versus PLHIV-T calculated based on temperatures predicted by SSP245.

Year	Scenario	PLHIV due to temperature increases (%)	Annual incident cases attributable to temperature increases (Percentage of annual incident cases [range])	Resulting cost of ART (US$ millions) (%)
2000	*Based on observed temperature* ^*1*^	1712 (0.05)	162 0.03 [0.03–0.03]	*Not *Applicable*
	*Based on temperatures per SSP245* ^*2*^	1339 (0.04)	162 0.03 [0.03–0.03]	*Not *Applicable*
	***Averted*** ***(%)***	**373** **(21.79)**		*Not *Applicable*
2005	*Based on observed temperature* ^*1*^	5291 (0.12)	447 0.09 [0.05–0.12]	0.15 (0.12)
	*Based on temperatures per SSP245* ^*2*^	4000 (0.09)	397 0.08 [0.05–0.10]	0.11 (0.09)
	***Averted*** ***(%)***	**1291** **(24.40)**		0.04 (24.40)
2010	*Based on observed temperature* ^*1*^	9195 (0.16)	472 0.12 [0.06–0.18]	2.07 (0.16)
	*Based on temperatures per SSP245* ^*2*^	6924 (0.12)	590 0.15 [0.07–0.16]	1.56 (0.12)
	***Averted*** ***(%)***	**2271** ***(*** **24.70)**		**0.51** ** (24.55)**
2015	*Based on observed temperature* ^*1*^	11,695 (0.18%)	409 0.14 [0.07–0.20]	2.89 (0.18)
	*Based on temperatures per SSP245* ^*2*^	8787 (0.13)	525 0.18 [0.10–0.19]	2.17 (0.13)
	***Averted*** ***(%)***	**2908** **(24.86)**		0.72 (24.86)
2020	*Based on observed temperature* ^*1*^	13,254 (0.18)	296 0.15 [0.08–0.22]	2.02 (0.18
	*Based on temperatures per SSP245* ^*2*^	9951 (0.13)	355 0.18 [0.13–0.23]	1.51 (0.13)
	***Averted*** ***(%)***	**3303** **(21.94)**		**0.51** ** (25.50)**
2025	*Based on temperatures per SSP245* ^*2*^	10,823 (0.14)	291 0.19 [0.14–0.24]	1.58 (0.14)
2035	*Based on temperatures per SSP245* ^*2*^	12,132 (0.15)	242 0.25 [0.20–0.30]	1.90 (0.15)
2050	*Based on temperatures per SSP245* ^*2*^	14,033 (0.20)	177 0.38 [0.30–0.42]	2.25 (0.20)

**Fig. 2:**
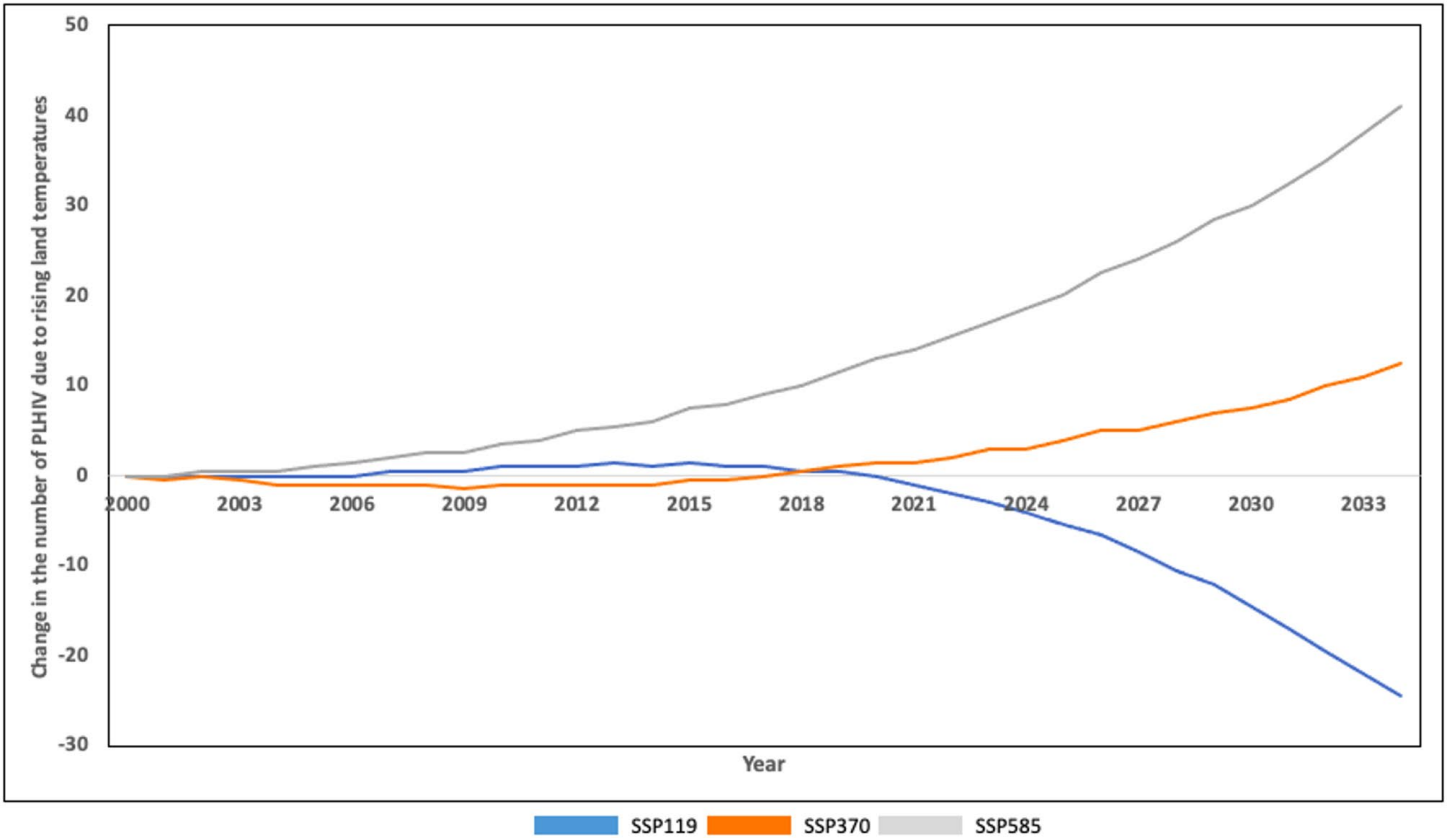
Change in the number of PLHIV due to land temperatures if shared socioeconomic pathway changed from SSP245 to SSP119 or SSP370 or SSP585 (between 2000–2050). Temperature predictions for the SSP scenarios are from the MAGICC7.0 model^14^. The PLHIV were calculated by extending methods and results reported by Baker^7^. The predicted number of PLHIV, which was used for the calculation, is from the Thembisa model^24^.

If temperatures continued as predicted by the SSP2 trajectory from 2021 to 2050, then by 2050 temperatures would increase by 1.98 °C above pre-industrial levels (to 18.53 °C), an increase of 0.60 °C from 2025 to 0.87 °C from 2000. The resulting increase in PLHIV-T ranges between 0.14% and 0.20% (mean = 0.16%; std. deviation = 0.02%). This means that in 2035, of the 8.10 million PLHIV, 12,132 [7,146 − 17,116] (0.15%) may be associated with warming. We estimated that the contribution to new HIV cases would be 0.25% (0.20–0.30). Of the PLHIV-T, 10,351 would access ART at a cost of US$1.90 million, assuming the predicted treatment coverage. In 2050, there would be 6.90 million PLHIV, with 14,033 [8,267 − 19,799](0.20%) due to warming. The contribution towards new HIV cases would be 0.38% (0.30–0.42); 12,237 would access ART at a cost of US$2.25 million. (Fig. 2; Table 2). SSP2 approximates other shared socioeconomic pathways in terms of PLHIV-T and their ART costs. We estimated that SSP119 however, would result in 25 fewer cases compared to SSP2 whereas SSP3 would result in 13 more cases and SSP5 would result in 41 more cases. The resulting excess follow-up ART costs in 2050 are therefore marginal (< US$7000) (appendix).

## Discussion

Unlike the number of PLHIV which has more than doubled from 2000 to 2020 (from 3.23 million to 7.45 million), the number of PLHIV-T has increased by more than seven-fold. If both climate policy and societal development remained unchanged from 2020 to 2050, this figure would further increase by nearly 41% even though HIV prevalence and incidence steadily decline. Alternatively, if climate policy remained unchanged but societal development switched from SSP2 to the more sustainable SSP1, then 139 fewer cases would result between 2020 and 2050 (Fig. 2) – a marginal reprieve considering the full cost avoidance possible. This suggests that without climate mitigation efforts, even optimistic societal development trajectories will have limited capacity to offset temperature-driven increases. Furthermore, all SSPs predict temperatures that are lower than recorded for 2000 to 2020 (Fig. 1). Therefore, the figures that we estimate are presumably a lower bound of excess HIV cases and costs that are possible.

Our results emphasize the urgency of climate action. Though estimated spending on ART represents a fraction of costs HIV-related costs^34,35^, other infections that are either opportunistic such as tuberculosis or that are synergistic such as other sexually transmitted infections^36^ add to these costs. Non-communicable diseases (NCDs) that are comorbid with HIV, especially those that have a climate association, further elevate them. Indeed, unlike HIV, tuberculosis incidence has been on the rise in South Africa since 2000^37^. Our estimates also present only the penalty due to global warming but not the penalty due to other climate events such as excess precipitation^10^, flooding or droughts^12^, all of which are known to be associated with HIV transmission, as well as their second order effects such as reductions in agricultural yield^38^, income shocks^14^ and food insecurity^39^. This study should therefore also be understood in the broader context of syndemic theory^40^ where climate stressors intersect with existing epidemics, poverty, food insecurity, and migration. South Africa’s HIV epidemic cannot be viewed in isolation; instead, a climate justice lens is essential to understand how structural inequities amplify health vulnerabilities.

Our estimates have several important implications. Currently known estimates of macroeconomic damages due to climate risks in South Africa are based on macro-structural models that do not include damage functions for health^41^. The penalty that we quantified therefore supplements the currently known damage figure of 1.5 trillion South African Rand (approximately US$94 billion), which excludes health impacts^42^. By placing a price tag in terms of HIV, our estimates also allow for a more direct inclusion of HIV in Nationally Determined Contributions. As of 2020, of 119 Nationally Determined Contributions submitted, only one country, Malawi, highlighted HIV as a priority issue for climate change management^43^. Our estimates also provide a starting point for assessing the additional co-financing needed for HIV programs to counter excess incidence due to climate effects. This is especially important for South Africa where domestic sources currently account for approximately 91% of annual climate finance^18^. Our estimates also have implications for the achievement of the 95-95-95 targets and the likelihood of ending AIDS as a public health threat by 2030^44^. HIV incidence was expected to steadily decline through 2100, largely due to our assumption of continued high coverage of treatment and general population testing^45^. Even if incidence were to continue to decline, despite the cessation on PEPFAR-funded HIV support, scaling back general population testing as several countries have done [46] risks a resurgence, especially if climate events disrupt public health infrastructure.

Our study has limitations. Principal among them is the reliance on the regression framework proposed by Baker, which also does not account for the pathways linking surface temperature increases to HIV prevalence^29^. Additionally, our estimates of land temperature increases were calculated assuming the pre-industrial baseline to be 1857, the earliest year for which data is available for South Africa, rather than 1850 as is customary. We assumed the impact of this adjustment to be minimal. The temperatures from 2021 to 2050 predicted for the SSPs were not available subnationally as they were not specific to South Africa. Therefore, we could not account for the subnational variation of prevalence. Since 1990, the national average temperature in South Africa has increased at a rate more than twice that of global temperature increases^47^. Therefore, our estimates likely underestimate the HIV penalty. However, our model does not account for adaptation mechanisms, which has the potential to overestimate the climate penalty. We assumed that ART unit costs would remain unchanged after 2025 other than adjusting for inflation, due to the difficulty in accounting for the uncertainty in future drug prices and staff salaries. In our sensitivity analysis, we considered the scenario where the decline in unit costs follow the per capita GDP even though historically this has not been the case^48^.

Even if not inclusive of all costs and impacts, our estimates suggests that climate change exerts a measurable burden on HIV incidence and care costs. Global temperatures have already surged past the 1.54 °C Paris Agreement target and if the NCQG indeed falls short, then mechanisms such as the Loss and Damage Fund must be operationalized promptly to mitigate funding shortfalls. Such action will undoubtedly have positive exernalities for the healthcare system as a whole. By quantifying a “climate penalty” on HIV, this analysis adds to the call for climate finance to incorporate health sector resilience as a core objective.

## Supplementary Information

Below is the link to the electronic supplementary material.

Supplementary Material 1

## Data Availability

Data generated or analyzed during this study are included in the main text and the supplementary material. The model codes programmed in STATA can be obtained by contacting the corresponding author at sas7443@mail.harvard.edu.
