# Supplementary material for: Estimated HIV cases and costs in South Africa due to global warming from 2000 to 2050: a modeling study
Source: Sci Rep. 2026 Jul 14;16:23460. doi: 10.1038/s41598-026-57530-1 (PMC13416121; doi:10.1038/s41598-026-57530-1)
Supplement: Supplementary file 1 — Supplementary Material 1 [file 41598_2026_57530_MOESM1_ESM.docx]

**Estimated HIV Cases and Costs in South Africa due to Global Warming from 2000 to 2050 – A Modeling Study**

**Supplementary Appendix**

SILVA, Sachin^1^, MEYER-RATH, Gesine^2,3^, ATUN, Rifat^4^, REID, Michael JA^5^

1. Institute for Global Health Sciences, University of California San Francisco

550 16th Street San Francisco, CA 94158, USA

1. Health Economics and Epidemiology Research Office (HE2RO)

University of the Witwatersrand

Princess of Wales Terrace, Parktown, Johannesburg, South Africa, 2193

1. Boston University School of Public Health

715 Albany St, Boston, MA 02118, USA

1. Harvard TH Chan School of Public Health, Harvard University

677 Huntington Avenue, Boston, MA 02115, USA

1. University of California, San Francisco, School of Medicine

513 Parnassus Avenue, San Francisco, CA 94143-0410, USA

**Corresponding author:**

Sachin Silva, DrPH

Institute for Global Health Sciences, University of California San Francisco

550 16th Street, San Francisco, CA 94158, USA

Email: SAS7443@mail.harvard.edu

**Methods**

*Land temperature projections in South Africa*

We extracted land temperatures for South Africa from 2000 to 2020 from Berkeley Earth[1]. These temperatures were reported in Celsius as monthly, annual, five-year, ten-year and twenty-year anomalies relative to a January 1951-December 1980 average annual or monthly temperature reference. For annual temperatures, this reference was Celsius (^0^C) 17.43 +/-0.19. The uncertainties that were reported represented the 95% confidence interval for statistical noise and spatial under sampling effects[1]. For the 2020 to 2050 period, we used global rather than South Africa specific temperatures that were reported for each shared socioeconomic pathway (SSP)[2] based on projections from the Model for the Assessment of Greenhouse Gas Induced Climate Change (MAGICC7), which is a prime reduced-complexity model[3], often used by IPCC for scientific publications and also by a number of Integrated Assessment Models (IAMs). To our knowledge, South Africa specific temperatures are not projected for each SSP.

| ***Year*** | ***Monthly Anomaly*** | ***Monthly Uncertainty*** | ***Annual Anomaly*** | ***Annual Uncertainty*** | ***Anomaly Reference*** | ***Calculated***  ***Temperature*** | ***Monthly Temperature (LB)*** | ***Monthly Temperature (UB)*** | ***Mean Monthly Temperature*** |
| --- | --- | --- | --- | --- | --- | --- | --- | --- | --- |
| 2000 | -1.384 | 0.2 | 0.38 | 0.076 | 22.81 | 21.43 | 21.03 | 21.83 | 17.66 |
| 2001 | 0.115 | 0.154 | 0.576 | 0.05 | 22.81 | 22.92 | 22.57 | 23.28 | 17.96 |
| 2002 | -0.068 | 0.219 | 0.453 | 0.085 | 22.81 | 22.74 | 22.32 | 23.16 | 18.04 |
| 2003 | 1.157 | 0.328 | 0.958 | 0.128 | 22.81 | 23.97 | 23.44 | 24.50 | 18.38 |
| 2004 | 0.553 | 0.253 | 0.63 | 0.111 | 22.81 | 23.36 | 22.91 | 23.82 | 18.27 |
| 2005 | 0.747 | 0.188 | 1.061 | 0.136 | 22.81 | 23.56 | 23.17 | 23.94 | 18.43 |
| 2006 | 0.494 | 0.343 | 0.555 | 0.166 | 22.81 | 23.30 | 22.76 | 23.85 | 17.82 |
| 2007 | 1.186 | 0.135 | 0.806 | 0.105 | 22.81 | 24.00 | 23.66 | 24.33 | 18.17 |
| 2008 | 0.082 | 0.175 | 0.566 | 0.101 | 22.81 | 22.89 | 22.52 | 23.27 | 18.16 |
| 2009 | 0.474 | 0.122 | 0.737 | 0.068 | 22.81 | 23.28 | 22.96 | 23.61 | 18.07 |
| 2010 | 0.345 | 0.274 | 0.95 | 0.078 | 22.81 | 23.16 | 22.68 | 23.63 | 18.53 |
| 2011 | 0.081 | 0.226 | 0.626 | 0.153 | 22.81 | 22.89 | 22.47 | 23.32 | 17.73 |
| 2012 | 1.304 | 0.156 | 0.491 | 0.147 | 22.81 | 24.11 | 23.76 | 24.47 | 17.97 |
| 2013 | 0.72 | 0.266 | 0.653 | 0.13 | 22.81 | 23.53 | 23.06 | 24.00 | 18.04 |
| 2014 | 1.132 | 0.269 | 0.48 | 0.136 | 22.81 | 23.94 | 23.47 | 24.41 | 18.16 |
| 2015 | 1.086 | 0.163 | 0.942 | 0.089 | 22.81 | 23.90 | 23.53 | 24.26 | 18.88 |
| 2016 | 1.793 | 0.322 | 1.689 | 0.137 | 22.81 | 24.60 | 24.08 | 25.13 | 18.89 |
| 2017 | -0.012 | 0.298 | 1.263 | 0.116 | 22.81 | 22.80 | 22.30 | 23.30 | 18.30 |
| 2018 | 1.036 | 0.19 | 0.845 | 0.077 | 22.81 | 23.85 | 23.46 | 24.24 | 18.38 |
| 2019 | 1.01 | 0.255 | 1.299 | 0.106 | 22.81 | 23.82 | 23.36 | 24.27 | 19.07 |
| 2020 | 0.794 | 0.213 | 1.191 | 0.078 | 22.81 | 23.60 | 23.19 | 24.02 | 18.20 |

**Table 1: Reported monthly and annual land temperature calculation based on reporting from Berkeley Earth[1].** The uncertainty reference is assumed to be 0.20.

*Incident and prevalent HIV cases in South Africa*

To estimate incident and prevalent HIV cases, and the share receiving antiretroviral therapy (ART) through 2050, we used outputs from the Thembisa transmission model developed by Johnson et al. which has been described elsewhere[4]. The model divided the population into cohorts defined in terms of demographic characteristics such as age, sex, marital status, behavioral characteristics such as sexual experience, propensity for commercial sex, concurrent partnerships, as well as level of exposure to HIV prevention programs, with individuals classified according to their HIV testing history[5], current receipt of pre-exposure prophylaxis (PrEP)/microbicides, and in the case of men, circumcision status. HIV-positive individuals were further classified according to their receipt of HIV care, as undiagnosed, diagnosed but untreated, and treated, and CD4 cell count[4].

*PLHIV due to land temperature increases*

Following Baker[6], we calculated the PLHIV that can be attributed to the effects of rising land temperatures, taking into account HIV prevalence $\left( HIV_{t} \right)$ and ART coverage $\left( A_{t} \right)$predicted by the Thembisa transmission model. The coefficient representing the estimated effect of temperature of HIV prevalence $(\beta_{P})$ was estimated by Baker to be 0.00477 (std. error = 0.00196) for a 1^0^C change in average monthly temperatures over a 6-year period, given a baseline prevalence of 5.6%[6]. We recalculated this coefficient $(\beta_{P})$ for each year’s prevalence and ART coverage and applied it per Baker’s specification[6]. As this calculation depended on prior period’s prevalence, it was done recursively.

| ***Year*** | ***Temperature*** | ***ART coverage*** | ***Coefficient*** | ***Coefficient LB*** | ***Coefficient UB*** |
| --- | --- | --- | --- | --- | --- |
| 2000 | 21.426 | 0.00 | 0.102 | 0.098 | 0.106 |
| 2001 | 22.925 | 0.00 | 0.109 | 0.105 | 0.113 |
| 2002 | 22.742 | 0.01 | 0.108 | 0.104 | 0.112 |
| 2003 | 23.967 | 0.01 | 0.113 | 0.110 | 0.117 |
| 2004 | 23.363 | 0.01 | 0.110 | 0.106 | 0.114 |
| 2005 | 23.557 | 0.03 | 0.109 | 0.105 | 0.113 |
| 2006 | 23.304 | 0.06 | 0.105 | 0.101 | 0.109 |
| 2007 | 23.996 | 0.10 | 0.104 | 0.100 | 0.107 |
| 2008 | 22.892 | 0.14 | 0.093 | 0.090 | 0.097 |
| 2009 | 23.284 | 0.20 | 0.089 | 0.085 | 0.093 |
| 2010 | 23.155 | 0.26 | 0.082 | 0.078 | 0.085 |
| 2011 | 22.891 | 0.34 | 0.073 | 0.069 | 0.076 |
| 2012 | 24.114 | 0.44 | 0.064 | 0.060 | 0.068 |
| 2013 | 23.53 | 0.51 | 0.056 | 0.052 | 0.059 |
| 2014 | 23.942 | 0.54 | 0.052 | 0.048 | 0.056 |
| 2015 | 23.896 | 0.58 | 0.048 | 0.044 | 0.052 |
| 2016 | 24.603 | 0.62 | 0.045 | 0.041 | 0.049 |
| 2017 | 22.798 | 0.66 | 0.037 | 0.033 | 0.041 |
| 2018 | 23.846 | 0.69 | 0.035 | 0.031 | 0.039 |
| 2019 | 23.82 | 0.72 | 0.032 | 0.028 | 0.036 |
| 2020 | 23.604 | 0.75 | 0.029 | 0.025 | 0.033 |

**Table 2: Annual temperature and corresponding excess risk of HIV prevalence.** Calculated based on methods described by Baker, using annual land temperatures as well as ART coverage rates.

*Incident HIV due to land temperature increases*

We derived an HIV incidence (per 100 person-years at risk and as a percent of at-risk population) that would give rise to the increase in prevalence due to land temperature increases, using two methods described by Hallett et al.[7]. One of these methods used cohort mortality rates and the other used information on survival after infection[7]. In the calculation, we treated the age specific cross-sectional prevalence without the contribution of rising land temperatures as the first serosurvey round and the cross-sectional prevalence with the temperature contribution as the second serosurvey round. We used mortality rates derived based on mortality projected by the Thembisa model[4]. We reported the age averaged values of incidence as a percent of at-risk population with lower and upper bounds corresponding to lower and upper bounds of the coefficient representing the estimated effect of temperature of HIV prevalence $\left( \beta_{P} \right) (Table 2)$.

| ***Age group*** | ***Cohort mortality rate*** |
| --- | --- |
| 10 | 0.0142 |
| 15 | 0.0004 |
| 20 | 0.0002 |
| 25 | 0.0006 |
| 30 | 0.0014 |
| 35 | 0.0022 |
| 40 | 0.0028 |
| 45 | 0.0028 |
| 50 | 0.0034 |
| 55 | 0.0048 |
| 60 | 0.0071 |
| 65 | 0.0104 |
| 70 | 0.0162 |
| 75 | 0.0237 |
| 80 | 0.0368 |
| 85 | 0.0557 |
| 90 | 0.0812 |

**Table 3: Cohort mortality rates assumed for each age group by type of epidemic**. Values are derived based on mortality predicted by the Thembisa model[4].

| ***Year*** | ***Incident infections*** | ***Incident infections attributable to temperature increases***  ***(percent)*** |
| --- | --- | --- |
| 2000 | 538,626 | 161  (0.03) |
| 2005 | 496,855 | 447  (0.09) |
| 2010 | 393,096 | 471  (0.12) |
| 2015 | 291,821 | 525  (0.18) |
| 2020 | 197,132 | 295  (0.15) |
| 2025 | 152,949 | 290  (0.19) |
| 2035 | 96,645 | 241  (0.25) |
| 2050 | 46,494 | 176  (0.38) |

**Table 4: Incident infections by year – Total and share attributable to surface temperature increases (2000-2050)**. Values are derived using methods described by methods described by Hallett et al.[7]

*ART cost calculation*

Our estimates of the excess cost of providing follow-up ART due to rising land temperatures were limited to the period from 2005 to 2050. For 2005, we used per patient per year costs reported by Cleary et al.[8] for public sector sites. For 2010 and 2015, we used unit costs reported by Meyer-Rath et al.[9]. For 2020, we used spending on ART per patient per year reported in South Africa’s National AIDS Spending Assessment 2017/18-2019/20[10]. For 2025, we used unit costs reported by Jamieson and colleagues[11] for 2019-2025. For 2030 and 2035, we assumed that unit costs remain unchanged from 2025 values, considering the difficulty in accounting for uncertainty in future drug prices, staff salaries, inflation etc. We validated these unit costs against unit costs derived by dividing the total expenditure on treatment by the number of individuals receiving treatment, from UNAIDS AIDSInfo (where possible)[12].

| ***Year*** | ***Unit cost (per patient per year)*** | ***Source*** |
| --- | --- | --- |
| 2005 | 1252.11 | Cleary et al. [8] |
| 2010 | 1129.89 | Meyer-Rath et al. [9] |
| 2015 | 488.34 | Meyer-Rath et al. [9] |
| 2020 | 211.11 | South Africa NASA 2017/18-2019/20[10] |
| 2025 | 184.01 | Jamieson et al. [11] |
| 2035 | 184.01 | Jamieson et al. [11] |
| 2050 | 184.01 | Jamieson et al. [11] |

**Table 5: Assumed per patient per year cost of providing follow-on ART in South Africa.** All costs reported in 2024 US$ rates. Unit costs are assumed to remain invariant from 2025 onwards.

*Sensitivity Analysis*

We conducted a probabilistic sensitivity analysis to evaluate the sensitivity of our estimates of PLHIV whose infection status can be associated with surface temperature increases and their ART costs. We assumed that these annual risks were normally distributed with a standard deviation similar to that of the original point estimate reported by Baker, which we calculated based on the reported standard errors and the number of observations[6]. From the respective distribution for each year, we sampled 5000 draws using a Latin Hypercube Sampling algorithm[13]. This sensitivity analysis was performed using Stata (IC version 14.2) and Microsoft Excel for Mac (version 16.45).

| ***Parameter*** | ***Year*** | ***Upper and Lower Bound*** | ***Additional people living with HIV due to temperature increase*** | ***Additional cost of ART due to temperature increase (US$)*** |
| --- | --- | --- | --- | --- |
| Coefficient representing the effect of temperature on HIV prevalence ($\beta$) | 2005 | 0.0747 - 0.0824 | 2, 762 - 7, 968 | 76,992 – 222,139 |
|  | 2010 | 0.0548 - 0.0624 | 5, 772 - 12, 942 | 1.30 mil – 2.92 mil |
|  | 2015 | 0.0408 - 0.0485 | 5, 946 - 16, 534 | 1.47 mil – 4.09 mil |
|  | 2020 | 0.0266 - 0.0343 | 7, 088 - 17, 998 | 1.91 mil – 2.93 mil |
|  | 2025 | 0.0255 -0.0332 | 6, 122 - 15, 280 | 0.89 mil – 2.22 mil |
|  | 2035 | 0.0222 -0.0299 | 5, 632 - 19, 466 | 0.88 mil – 3.06 mil |
|  | 2050 | 0.0229 - 0.0306 | 9, 086 - 19, 594 | 1.46 mil – 3.14 mil |

**Table 6: Sensitivity to uncertainty in the excess risk of HIV prevalence due to land temperature increase.** All other parameters were assumed to be remain invariant.

**
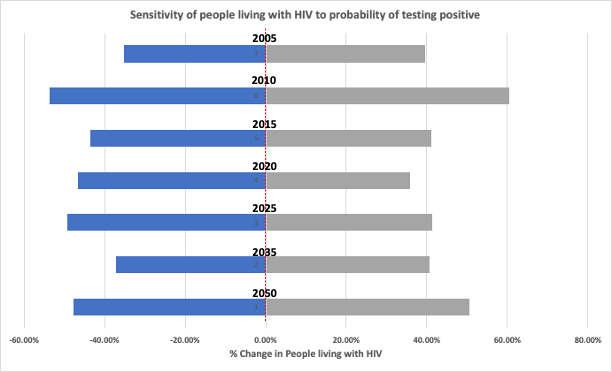
**

**Figure 1: Tornado diagram representing sensitivity to uncertainty in the excess risk of HIV prevalence due to land temperature increase.** All other parameters were assumed to be remain invariant.

*Currency conversion*

When monetary amounts were reported in international dollars (I$), we applied the purchasing power parity rates (PPP) to first convert to local currency units (LCUs) for the same year, then applied the appropriate GDP deflators to inflate/deflate to 2024 LCUs, then converted the 2024 LCUs to 2024 USDs using the official exchange rates. When amounts were reported in LCUs for a year other than 2020, we first inflated/deflated to 2024 using the appropriate deflators, then converted to 2024 US$ rates by using the corresponding exchange rates. When amounts were reported in USDs for a year other than 2024, we used the deflators to inflate/deflate to 2024 as needed. Deflator values, PPP rates and exchange rates were extracted from the World Bank[14–16].

**References**

1. Berkeley Earth. Berkeley Earth - South Africa [Internet]. Berkeley Earth. 2025 [cited 2025 May 29]. Available from: https://berkeleyearth.org/temperature-region/

2. Riahi K, van Vuuren DP, Kriegler E, Edmonds J, O’Neill BC, Fujimori S, et al. The Shared Socioeconomic Pathways and their energy, land use, and greenhouse gas emissions implications: An overview. Global Environmental Change [Internet]. 2017 Jan 1 [cited 2024 Apr 1];42:153–68. Available from: https://www.sciencedirect.com/science/article/pii/S0959378016300681

3. Meinshausen M, Raper SCB, Wigley TML. Emulating coupled atmosphere-ocean and carbon cycle models with a simpler model, MAGICC6 – Part 1: Model description and calibration. Atmospheric Chemistry and Physics [Internet]. 2011 Feb 16 [cited 2024 Mar 30];11(4):1417–56. Available from: https://acp.copernicus.org/articles/11/1417/2011/

4. Johnson LF, Chiu C, Myer L, Davies MA, Dorrington RE, Bekker LG, et al. Prospects for HIV control in South Africa: a model-based analysis. Glob Health Action [Internet]. 2016 Jun 8 [cited 2024 Sep 14];9:10.3402/gha.v9.30314. Available from: https://www.ncbi.nlm.nih.gov/pmc/articles/PMC4901512/

5. Johnson LF, Rehle TM, Jooste S, Bekker LG. Rates of HIV testing and diagnosis in South Africa: successes and challenges. AIDS. 2015 Jul 17;29(11):1401–9.

6. Baker RE. Climate change drives increase in modeled HIV prevalence. Climatic Change [Internet]. 2020 Nov 1 [cited 2024 Dec 25];163(1):237–52. Available from: https://doi.org/10.1007/s10584-020-02753-y

7. Hallett TB, Zaba B, Todd J, Lopman B, Mwita W, Biraro S, et al. Estimating Incidence from Prevalence in Generalised HIV Epidemics: Methods and Validation. PLoS Med [Internet]. 2008 Apr [cited 2024 Dec 25];5(4):e80. Available from: https://www.ncbi.nlm.nih.gov/pmc/articles/PMC2288620/

8. Cleary SM, McIntyre D, Boulle AM. The cost-effectiveness of antiretroviral treatment in Khayelitsha, South Africa--a primary data analysis. Cost Eff Resour Alloc. 2006 Dec 6;4:20.

9. Meyer-Rath G, Johnson LF, Pillay Y, Blecher M, Brennan AT, Long L, et al. Changing the South African national antiretroviral therapy guidelines: The role of cost modelling. PLOS ONE [Internet]. 2017 Oct 30 [cited 2025 May 1];12(10):e0186557. Available from: https://journals.plos.org/plosone/article?id=10.1371/journal.pone.0186557

10. he South African National AIDS Council (SANAC). National AIDS Spending Assessment 2017/18-2019/20 [Internet]. he South African National AIDS Council (SANAC); [cited 2025 May 29]. (National AIDS Spending Assessment). Available from: https://sanac.org.za/reports/sa-nasa/

11. Jamieson L, Johnson LF, Nichols BE, Delany-Moretlwe S, Hosseinipour MC, Russell C, et al. Relative cost-effectiveness of long-acting injectable cabotegravir versus oral pre-exposure prophylaxis in South Africa based on the HPTN 083 and HPTN 084 trials: a modelled economic evaluation and threshold analysis. The Lancet HIV [Internet]. 2022 Dec 1 [cited 2024 Sep 13];9(12):e857–67. Available from: https://www.thelancet.com/journals/lanhiv/article/PIIS2352-3018(22)00251-X/fulltext

12. AIDSinfo | UNAIDS [Internet]. [cited 2019 May 12]. Available from: http://aidsinfo.unaids.org/

13. Orwa TO, Mbogo RW, Luboobi LS. Uncertainty and Sensitivity Analysis Applied to an In-Host Malaria Model with Multiple Vaccine Antigens. Int J Appl Comput Math [Internet]. 2019 May 24 [cited 2021 Apr 9];5(3):73. Available from: https://doi.org/10.1007/s40819-019-0658-3

14. World Bank, Washington, DC. World Bank Open Data - Official Exchange Rates [Internet]. World Bank Open Data. [cited 2025 Jun 10]. Available from: https://data.worldbank.org

15. World Bank, Washington, DC. World Bank Open Data - GDP Deflators [Internet]. World Bank Open Data. [cited 2025 Jun 10]. Available from: https://data.worldbank.org

16. World Bank, Washington, DC. World Bank Open Data - Purchasing Power Parity Rates [Internet]. World Bank Open Data. [cited 2025 Jun 10]. Available from: https://data.worldbank.org
